# Between definitive resection and damage control observation: a case series analysis of intraoperative decision-making for portal venous gas

**DOI:** 10.3389/fsurg.2026.1853201

**Published:** 2026-06-24

**Authors:** Fengqi Ruan, Yongen Liu, Shuai Wang

**Affiliations:** 1 Department of Emergency Surgery, Linyi People's Hospital, Linyi, Shandong, China; 2 Department of Emergency, Linyi People's Hospital, Linyi, Shandong, China

**Keywords:** acute abdomen, acute mesenteric ischemia, damage control surgery, portal venous gas, surgical decision-making

## Abstract

**Objective:**

To investigate surgical decision-making in patients with portal venous gas (PVG) and acute abdomen, and to summarize the basis for choosing definitive resection vs. damage control observation according to intraoperative intestinal viability.

**Methods:**

We retrospectively analyzed 4 patients with PVG and acute abdomen who underwent exploratory laparotomy at Linyi People's Hospital between April 2024 and December 2025. Based on intraoperative bowel color, peristalsis, and terminal mesenteric arterial pulsation, patients were classified into a definitive resection group (confirmed transmural necrosis, *n* = 2) or a damage control observation group (extensive ischemia without definite transmural necrosis, *n* = 2).

**Results:**

All patients presented with acute abdominal pain, and preoperative CT showed PVG with intestinal wall abnormalities or peritoneal effusion. In the definitive resection group, necrosis involved either 40 cm of jejunum or 70 cm of ileum and was treated by resection and anastomosis. Recovery was uneventful, with a mean hospital stay of 8 days. In the damage control observation group, long segments of ischemic small bowel without definite necrosis were preserved after exploration, peritoneal lavage, and drainage. These patients developed more pronounced postoperative inflammatory and coagulation abnormalities. One had a D-dimer level of 5.45 μg/mL (FEU), and another with end-stage liver disease required ICU admission and a 20-day stay. All patients survived to discharge.

**Conclusion:**

Based on this small case series, PVG may indicate serious intestinal pathology in acute abdomen and supports timely exploration. Intraoperative bowel viability assessment may guide resection or preservation of borderline ischemic bowel. These exploratory findings require confirmation in larger studies.

## Introduction

Portal venous gas (PVG) has historically been considered closely associated with intestinal necrosis and extremely high mortality (1, 2). With the widespread use of computed tomography (CT), the detection rate of PVG has increased substantially, and its etiological spectrum has broadened. In addition to intestinal ischemia and necrosis, PVG may also occur in a variety of clinical settings, including inflammatory bowel disease, gastrointestinal perforation, and certain medical procedures (3, 4). Therefore, PVG itself is not an independent disease entity but rather an imaging finding that requires comprehensive evaluation based on its underlying etiology and the patient's clinical condition (3–5).

For patients with PVG complicated by acute abdomen, the key challenge in surgical management lies not in the presence of PVG *per se*, but in accurately determining the severity and reversibility of intestinal injury. In prior clinical practice, PVG was often regarded as an indirect indicator of intestinal necrosis, leading to a tendency toward aggressive surgical exploration or even extensive resection. However, several studies have demonstrated that a proportion of patients undergoing emergency surgery for suspected PVG-associated intestinal necrosis ultimately do not require bowel resection (5). Furthermore, the latest guidelines on acute mesenteric ischemia issued by the World Society of Emergency Surgery (WSES) explicitly emphasize that surgical management of borderline ischemic bowel should be highly individualized to avoid unnecessary over-resection (6). These findings highlight that surgical decision-making in PVG-related acute abdomen should rely more on direct intraoperative assessment of intestinal viability rather than solely on imaging findings.

When extensive intestinal ischemia without clear irreversible necrosis is encountered intraoperatively, whether to proceed with immediate resection of all affected bowel segments remains one of the most challenging decisions. In this context, the concept of damage control surgery (DCS) provides an alternative approach: by controlling contamination and minimizing operative trauma, surgeons aim to preserve potentially viable bowel and allow time for subsequent reassessment and supportive management (7). Based on this rationale, the present study retrospectively analyzed four patients with PVG complicated by acute abdomen who underwent emergency exploratory laparotomy. Focusing on intraoperative intestinal viability assessment, we summarized the applicable scenarios and postoperative course characteristics of two decision-making strategies, definitive resection and damage control observation. This study aims to provide clinical reference for individualized surgical management of PVG-related acute abdomen (Figures 1–3).

**Figure 1:**
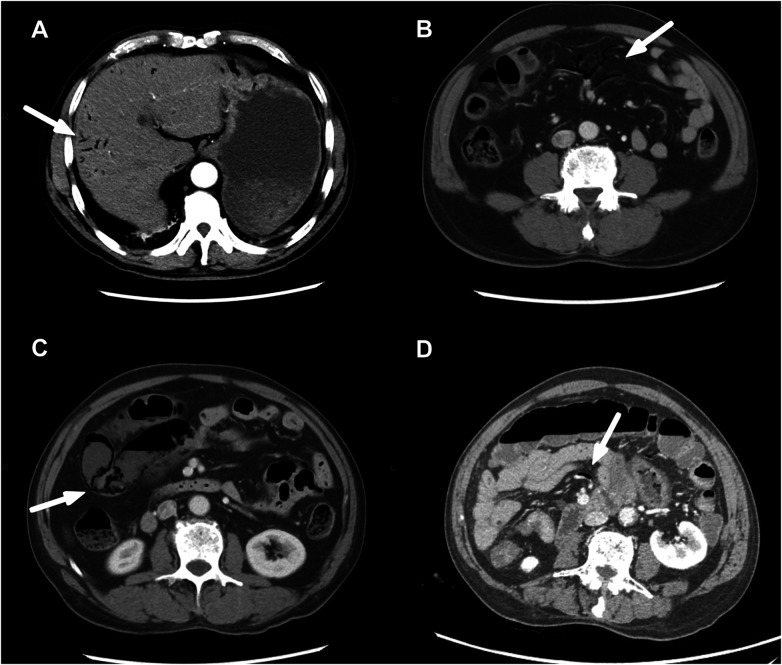
Representative preoperative CT images of four patients with PVG-associated acute abdomen. **(A)** Intrahepatic PVG. **(B)** Mesenteric venous gas. **(C)** Abnormal changes of the small intestinal wall. **(D)** Additional high-risk features, including suspected internal hernia/small bowel volvulus or intra-abdominal fluid.

**Figure 2:**
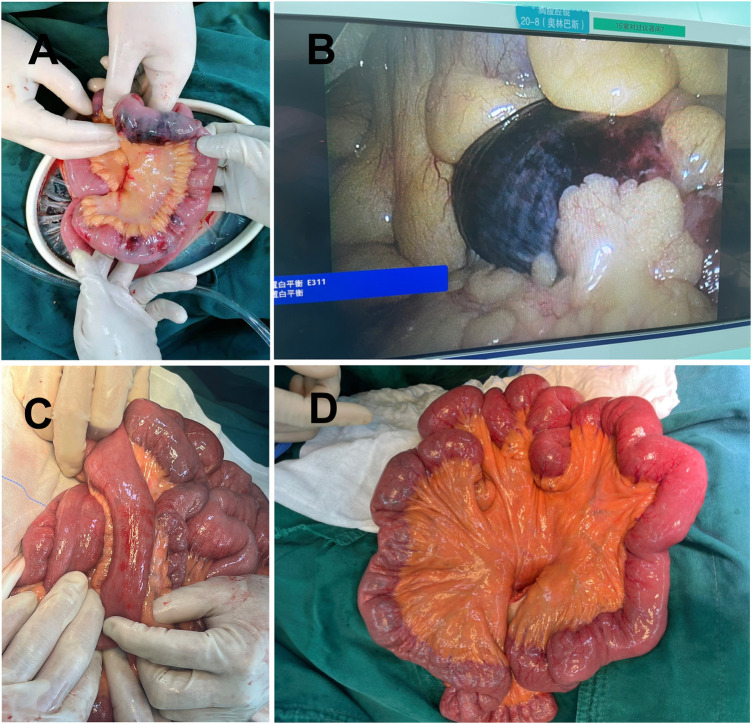
Representative intraoperative findings from the intestinal viability assessment. **(A,B)** Intestinal segments with definitive irreversible necrosis, characterized by dark purple-to-black discoloration, consistent with the definitive resection pathway. **(C,D)** Intestinal segments exhibiting extensive ischemia without definitive transmural necrosis, characterized by congestion, edema, and dusky discoloration, consistent with the damage control observation pathway.

**Figure 3:**
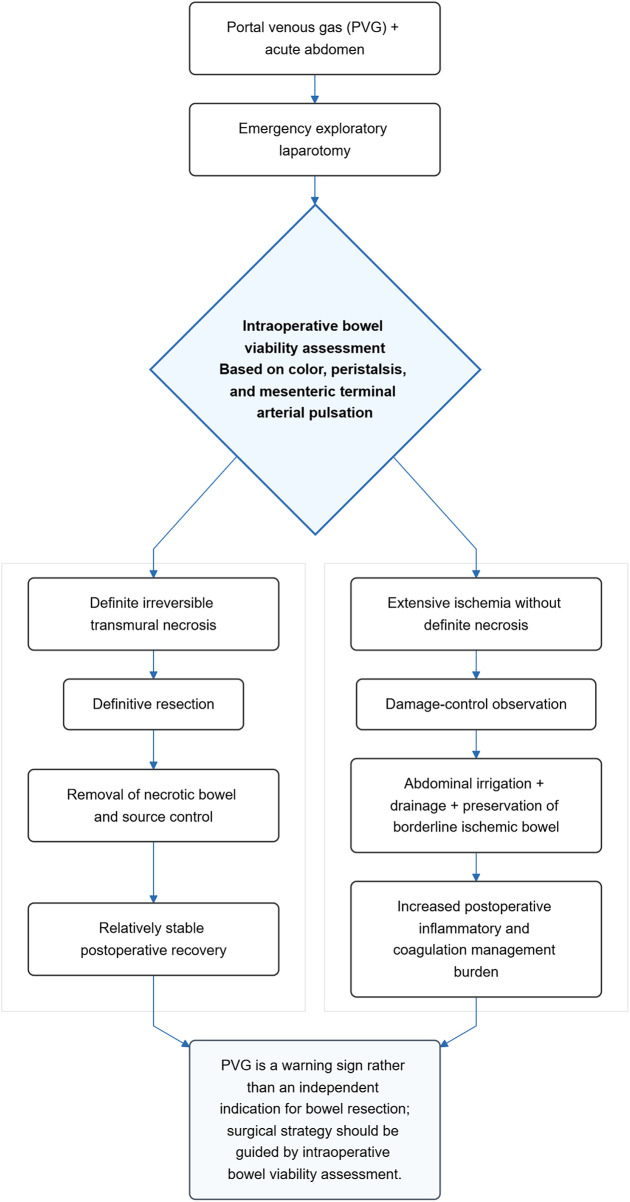
Schematic diagram illustrating intraoperative and postoperative decision-making pathways in patients with PVG-associated acute abdomen. Following emergency exploratory laparotomy, bowel viability was assessed using visual and tactile criteria, including bowel color, peristalsis, and terminal mesenteric arterial pulsation. When available, adjunctive objective perfusion tools, such as indocyanine green fluorescence angiography or Doppler assessment, may further support intraoperative viability evaluation. Definite irreversible transmural necrosis prompted definitive resection, whereas extensive ischemia without definite transmural necrosis led to bowel-preserving damage-control observation, including irrigation, drainage, primary abdominal closure, and close postoperative monitoring. On-demand re-exploration or second-look laparotomy should be considered when clinically indicated, such as in cases of progressive peritonitis, refractory sepsis, worsening lactate or acidosis, persistent shock, or other signs of clinical deterioration. ICU support should be individualized according to physiological reserve and systemic complications. PVG, portal venous gas; ICG, indocyanine green; ICU, intensive care unit.

## Methods

### Study population

This study was a single-center retrospective case series analysis. Clinical data were retrospectively reviewed from four patients diagnosed with PVG complicated by acute abdomen who underwent emergency exploratory laparotomy at Linyi People's Hospital between April 2024 and December 2025.

Inclusion criteria were as follows: (1) PVG confirmed by preoperative abdominal CT; (2) clinical presentation of acute abdomen; and (3) emergency surgical exploration performed.

Exclusion criteria included: (1) conservative management without surgery; (2) iatrogenic or traumatic PVG; and (3) significantly incomplete clinical records.

The study was approved by the Medical Ethics Committee of Linyi People's Hospital.

### Intraoperative intestinal viability assessment and decision pathway definition

All patients underwent emergency exploratory surgery under general anesthesia, predominantly via open laparotomy; in one case, initial laparoscopic exploration was converted to open surgery. Intraoperative intestinal viability assessment was based on the surgeon's comprehensive evaluation of bowel color, peristalsis, and terminal mesenteric artery pulsation. According to intraoperative determination of intestinal necrosis severity, surgical management was categorized into two primary decision pathways:

1. **Definitive resection pathway**: Patients exhibiting localized and clearly irreversible transmural necrosis underwent segmental intestinal resection.
2. **Damage-control observation pathway**: Patients with extensive intestinal ischemia but no definite irreversible transmural necrosis were managed by bowel preservation *in situ*, peritoneal irrigation, drainage, and close postoperative reassessment. The term “damage-control observation” refers to this DCS-inspired approach and does not imply the use of temporary abdominal closure or planned second-look laparotomy. Re-exploration was reserved for clinical deterioration, refractory sepsis, or progressive peritonitis.

### Data collection and outcome measures

Data were collected from the hospital's electronic medical record system and included the following: (1) demographic characteristics and major comorbidities; (2) preoperative clinical presentations, laboratory results, and imaging findings; (3) intraoperative findings, specifically regarding location, extent, and severity of intestinal ischemia and necrosis; (4) details of surgical procedures and postoperative clinical course, emphasizing changes in inflammatory and coagulation parameters, ICU support requirements, major complications, length of hospital stay, and final clinical outcomes.

### Statistical analysis

Given the small sample size (*n* = 4), a descriptive approach was employed to summarize patient data. Continuous variables were presented as exact values or ranges, and categorical variables as frequencies. No inferential statistical analyses were conducted.

## Results

### Case 1: definitive resection following confirmed ileal necrosis

A 67-year-old man presented to the emergency department with sudden-onset acute abdominal pain. Preoperative contrast-enhanced abdominal CT revealed extensive PVG accompanied by ischemic changes of the intestinal wall (Figure 1).

Due to rapid clinical deterioration and clear signs of acute abdomen, emergency exploratory laparotomy was performed. Intraoperatively, approximately 400 mL of peritoneal exudate was observed. Approximately 70 cm of ileum demonstrated transmural necrosis (Figure 2A), characterized by blackened intestinal walls, complete absence of peristalsis, and non-palpable pulsation of the corresponding terminal mesenteric arteries. Given these definitive indicators of irreversible necrosis, segmental intestinal resection was decisively performed. Postoperative pathology confirmed extensive congestion, edema, and transmural necrosis of the resected intestinal segment. The patient's systemic inflammatory burden rapidly decreased following surgery, and overall recovery was relatively smooth. Although postoperative calf intermuscular venous thrombosis occurred (suggesting a prothrombotic state related to ischemia-reperfusion injury), the patient recovered successfully with standardized anticoagulation and supportive care and was discharged on postoperative day 9.

### Case 2: damage control observation for extensive ileal ischemia without definite necrosis

A 77-year-old man presented to the emergency department with acute abdominal pain lasting approximately 7 h. Preoperative abdominal CT suggested disordered small intestinal loops in the left abdomen, indicating possible internal hernia or small bowel volvulus, accompanied by suspected intramural gas, mesenteric venous gas, intrahepatic PVG, and perihepatic fluid (Figure 1). Emergency exploratory laparotomy identified approximately 500 mL of peritoneal exudate and approximately 150 cm of extensively ischemic small intestine (Figure 2C), characterized by congestion, edema, and decreased viability, but without definite signs of irreversible transmural necrosis. Considering the extensive involvement of the bowel and the risk of short bowel syndrome due to unnecessary extensive resection, a damage control observation strategy was adopted: abdominal exploration, peritoneal irrigation, and adequate drainage were performed, with preservation of the intestine *in situ*. Postoperatively, the patient exhibited pronounced inflammatory and coagulation abnormalities, including an elevated D-dimer of 5.45 μg/mL (FEU), accompanied by increased C-reactive protein (CRP) and fibrinogen levels, indicating a significant perioperative systemic response. The patient's condition gradually stabilized after comprehensive supportive treatment. Serial abdominal assessment and drainage monitoring revealed no progressive peritonitis. Laboratory follow-up did not suggest ongoing necrosis; re-exploration was therefore not undertaken.

### Case 3: damage control observation in a patient with end-stage liver disease and complicated postoperative course

A 75-year-old man with diabetes mellitus, alcoholic liver cirrhosis, portal hypertension, and esophagogastric varices presented with acute abdominal pain. Preoperative abdominal CT demonstrated intrahepatic gas, portal and mesenteric venous gas, edema of segments of the small intestinal walls, and a small amount of abdominopelvic fluid (Figure 1). Emergency exploratory laparotomy revealed extensive ischemia involving long segments of small intestine with markedly decreased viability (Figure 2D), but without definitive irreversible transmural necrosis. Considering the patient's poor baseline physiological status and the potentially severe consequences of extensive resection, a damage control observation approach was chosen, including abdominal exploration, peritoneal irrigation, and drainage, with bowel preservation. Postoperatively, the patient experienced a significantly complicated clinical course and was transferred to the intensive care unit (ICU), subsequently developing pulmonary infection, sepsis, and liver-related complications, accompanied by aggravated inflammatory and coagulation abnormalities. The D-dimer peaked at 3.50 μg/mL (FEU), and fibrinogen degradation products (FDP) increased to 10.20 μg/mL. Following comprehensive supportive care, the patient's condition gradually stabilized. Despite the systemic complications, abdominal findings remained stable, with no recurrent peritonitis or clinical evidence of delayed necrosis; re-exploration was not undertaken.

### Case 4: definitive resection for segmental small bowel necrosis

An 80-year-old woman presented with lower abdominal pain lasting one day. She had a medical history of hypertension and cerebral infarction. Preoperative abdominal CT demonstrated small intestinal wall thickening in the mid-to-lower abdomen, intramural gas, PVG, and abdominopelvic fluid (Figure 1). Emergency surgery was initially performed laparoscopically, revealing yellowish turbid ascites and a purple-black segment of small intestine (Figure 2B), prompting conversion to open laparotomy. Further exploration identified multifocal segmental necrosis of the small intestine from 15 to 55 cm distal to the ligament of Treitz, with thinning of the intestinal wall and surrounding edematous thickening. Segmental resection and end-to-end anastomosis of the small intestine were performed. Pathological examination of the resected specimen (55.5 cm in length) revealed congestion, hemorrhage, edema, infection, and necrosis, consistent with intestinal necrosis. The patient's postoperative recovery was relatively uneventful, and her condition stabilized following antimicrobial therapy and nutritional support.

Two distinct intraoperative decision-making pathways were established: Cases 1 and 4 underwent definitive resection due to confirmed irreversible transmural necrosis, whereas Cases 2 and 3 followed damage control observation due to extensive ischemia without definite necrosis. Baseline characteristics, intraoperative findings, surgical strategies, and clinical outcomes for each case are summarized in Table 1. Additional case-level preoperative CT findings are provided in Supplementary Figure S1. Key perioperative objective parameters and the postoperative support burden are presented in Table 2.

**Table 1:** Baseline characteristics, intraoperative findings, surgical strategies, and clinical outcomes of the 4 patients.

Case	Age/Sex	Major comorbidities	Main preoperative CT findings	Intraoperative findings	Surgical strategy	Major postoperative problems	Length of stay, (days)	Outcome
1	67/M	History of alcohol use	PVG; bowel wall thickening	Approximately 70 cm of ileum with definite necrosis	Ileal resection with primary anastomosis	Intermuscular venous thrombosis	9	Survived to discharge
2	77/M	History of smoking and alcohol use	PVG; mesenteric venous gas; suspected internal hernia or small-bowel volvulus; intra-abdominal fluid	Approximately 150 cm of small bowel with extensive ischemia but no definite necrosis	Exploratory laparotomy, abdominal irrigation, and drainage (damage-control observation)	Marked inflammatory and coagulation abnormalities	11	Survived to discharge
3	75/M	Diabetes mellitus; alcoholic cirrhosis; portal hypertension; gastroesophageal varices	PVG; portal and mesenteric venous gas; partial small-bowel wall edema; small amount of abdominopelvic fluid	Long-segment extensive small-bowel ischemia without definite necrosis	Exploratory laparotomy, abdominal irrigation, and drainage (damage-control observation)	ICU admission; pulmonary infection; sepsis; liver-related complications; worsening coagulation abnormalities	20	Survived to discharge
4	80/F	Hypertension; history of cerebral infarction	PVG; bowel wall thickening; abdominopelvic fluid	Multiple segmental areas of small-bowel necrosis, 15–55 cm distal to the ligament of Treitz	Partial small-bowel resection with primary anastomosis	Relatively uneventful recovery	7	Survived to discharge

PVG, portal venous gas; CT, computed tomography; ICU, intensive care unit.

“Damage-control observation” refers to *in situ* preservation of borderline ischemic bowel with irrigation and drainage, rather than immediate extensive resection. It was not intended as a standardized DCS protocol involving temporary abdominal closure or planned second-look laparotomy.

**Table 2:** Key perioperative objective indicators and postoperative support burden in the 4 patients.

Case	Decision pathway	Extent of involved bowel	Pathological confirmation of necrosis	Key coagulation/metabolic abnormalities	ICU support	Major postoperative critical problems	Length of stay, days
1	Definitive resection	Approximately 70 cm of ileum	Yes (73 cm ileal specimen; bowel wall congestion, hemorrhage, edema, and necrosis)	Peak D-dimer 1.75 μg/mL (FEU); fibrinogen 6.26 g/L	No	Intermuscular venous thrombosis	9
2	Damage-control observation	Approximately 150 cm of small bowel with extensive ischemia	No	Peak D-dimer 5.45 μg/mL (FEU); fibrinogen 4.33 g/L	No	Marked inflammatory and coagulation abnormalities	11
3	Damage-control observation	Long-segment extensive small-bowel ischemia	No	Peak D-dimer 3.50 μg/mL (FEU); FDP 10.20 μg/mL; peak lactate 4.9 mmol/L	Yes	ICU treatment, pulmonary infection, sepsis, liver-related complications, worsening coagulation abnormalities	20
4	Definitive resection	Multiple segmental necrotic lesions of the small bowel, 15–55 cm distal to the ligament of Treitz	Yes (55.5 cm small-bowel specimen; bowel wall congestion, hemorrhage, edema, infection, and necrosis)	No representative peak coagulation/metabolic value available from the current records	No	Relatively uneventful recovery	7

ICU, intensive care unit; FEU, fibrinogen-equivalent units; FDP, fibrin/fibrinogen degradation products.

“Damage-control observation” refers to *in situ* preservation of borderline ischemic bowel with irrigation and drainage, rather than immediate extensive resection. It was not intended as a standardized DCS protocol involving temporary abdominal closure or planned second-look laparotomy.

## Discussion

### Core considerations in surgical decision-making for PVG complicated by acute abdomen

This case series demonstrates that PVG itself is not an independent indicator for determining the surgical approach. Instead, surgical management should primarily rely on the direct intraoperative viability assessment and the identification of transmural necrosis. Although emergency exploration is frequently necessary for patients with PVG complicated by acute abdomen, intraoperative management is not a binary “resect or preserve” decision. Rather, it requires case-by-case judgment based on intestinal color, peristalsis, mesenteric artery pulsation, and the patient's physiological reserve. This viewpoint aligns with the 2022 WSES guidelines on acute mesenteric ischemia, which recommend limiting resection strictly to clearly necrotic bowel segments while preserving potentially viable segments whenever feasible (6).

In this study, patients identified intraoperatively as having definitive necrosis underwent immediate resection and experienced relatively uneventful postoperative recoveries. In contrast, patients with extensive ischemia but no definite necrosis were managed with a DCS-inspired bowel-preserving approach (7), which avoided immediate extensive resection and potential short bowel syndrome but imposed a more complex perioperative management course. A 2025 case series by Wei et al. further emphasized that etiological recognition, comorbidity assessment, and stratification of surgical indications are crucial for optimizing clinical management in PVG patients (8). These findings underscore that the clinical value of PVG lies more in its role as a warning sign rather than a decisive indication. Surgical decisions should therefore be guided primarily by real-time intraoperative assessment of intestinal viability rather than solely by preoperative imaging findings. The intraoperative pathway used in this study is illustrated in Figure 3.

### Decision watershed: balancing definitive resection and damage control observation

This case series indicates that the key intraoperative decision point in emergency exploration for PVG is whether definite, irreversible transmural intestinal necrosis is present. In cases involving localized segments of clearly necrotic intestine, prompt definitive resection effectively removes necrotic tissue and controls persistent pathological stimulation. However, for extensive ischemia without definitive necrosis, immediate extensive resection should be approached more cautiously.

Arai et al. reported that among adult PVG patients who underwent surgery for suspected intestinal necrosis, 47.1% ultimately did not require intestinal resection (5). This finding underscores that preoperative classification as high-risk does not invariably necessitate intraoperative bowel resection. Similarly, the 2022 WSES guidelines emphasize that surgical intervention should be limited to clearly necrotic segments, preserving potentially viable bowel whenever possible (6).

Against this backdrop, the introduction of the DCS concept (7) in patients with extensive ischemia and indeterminate intestinal viability is reasonable. The primary goal of DCS is not to “avoid surgery,” but rather to control peritoneal contamination, mitigate surgical stress, and gain time for further evaluation of borderline ischemic bowel segments. The 2025 JTACS algorithm for acute mesenteric ischemia suggests that a second-look procedure typically performed 12–24 h after initial surgery can reassess intestinal viability, with temporary abdominal closure providing a critical window for subsequent decision-making (9).

The approach used here should be separated from classical DCS. Temporary abdominal closure was not part of our routine pathway, and planned second-look laparotomy was not undertaken. Whether a planned second-look strategy would have changed the outcomes cannot be determined from these four cases.

However, existing evidence regarding the application of DCS in non-traumatic abdominal emergencies remains limited, and its precise role has yet to be fully defined (10, 11). Thus, employing this strategy should be based on individualized clinical judgment rather than adopted as routine practice.

### The double-edged sword of damage control strategy: local preservation vs. systemic inflammation-coagulation challenges

Although the DCS strategy allows time for borderline ischemic intestinal segments to recover, the present cases also highlight its potential “double-edged” effect. In this series, patients who did not undergo immediate intestinal resection (Cases 2 and 3), despite preserving intestinal continuity, exhibited more pronounced postoperative inflammation and coagulation abnormalities compared with the definitive resection group. This observation suggests that ischemic but unresected bowel segments may serve as a persistent pathological stimulus, triggering more complex systemic inflammatory cascades.

This clinical phenomenon of heightened systemic inflammation and coagulation abnormalities associated with local ischemic injury aligns with the emerging concept of “thromboinflammation” (12). Recent literature emphasizes that inflammation and coagulation are not isolated processes but rather mutually amplified interactions involving platelets, coagulation cascades, endothelial cells, and immune cells. During ischemia/reperfusion injury, the release of damage-associated molecular patterns (DAMPs) may further enhance tissue factor expression, thrombosis, and inflammatory responses. The inflammatory and coagulation abnormalities seen in Cases 2 and 3 may fit a thromboinflammatory response to ischemic bowel injury. This remains an indirect interpretation. No mechanistic biomarkers were available, so thromboinflammation should be viewed only as a possible explanatory framework.

Furthermore, a patient's baseline physiological reserve significantly influences tolerance to this strategy. Case 3, who had end-stage liver disease (cirrhosis), experienced a notably more complicated postoperative course compared with the other cases. According to the 2023 EASL guidelines for acute-on-chronic liver failure (ACLF), ACLF is driven by excessive systemic inflammatory responses and frequently associated with multiple organ dysfunction, necessitating early organ support and intensive care stabilization for critically ill patients (13). Therefore, when selecting a DCS strategy for patients with significant comorbidities, particularly those with immune fragility and coagulation dysregulation, it is essential to consider not only the benefit of intestinal preservation but also anticipate the increased systemic management burden. Intensive monitoring and robust supportive care strategies should thus be proactively planned.

### Advances and future prospects in intraoperative assessment techniques

The decision-making process in this case series highlights that, in current emergency surgical practice, decisions regarding the preservation or resection of intestinal segments continue to rely heavily on the surgeon's subjective assessment of intestinal color, peristalsis, and mesenteric artery pulsation. Such visual and tactile assessments are typically sufficient for clearly necrotic intestinal segments. However, in cases involving extensive ischemia with borderline viability, relying solely on these criteria remains inherently subjective. In the present cases, no objective perfusion assessment such as ICG fluorescence angiography or Doppler evaluation was available during emergency exploration. This remains an important limitation, particularly in patients with extensive borderline ischemia. Recently, intraoperative microcirculatory perfusion evaluation techniques, such as indocyanine green (ICG) fluorescence angiography, have been increasingly adopted in emergency surgery. The 2025 WSES international consensus statement recommends that, when equipment and technical resources are available, ICG may serve as an effective adjunctive tool for assessing intestinal perfusion and viability, thus supporting intraoperative decision-making in emergency scenarios (14). In the future, integration of such objective perfusion assessment tools into emergency exploration protocols for PVG and acute mesenteric ischemia could enhance the safety and consistency of decisions concerning intestinal resection vs. preservation.

## Limitations

This study has several limitations. First, it is a single-center retrospective case series with a small sample size (*n* = 4), limiting the generalizability of the findings. Second, intraoperative assessment of intestinal viability was based primarily on visual inspection and palpation, without routine use of objective perfusion assessment methods such as ICG fluorescence angiography. Third, continuous dynamic monitoring of inflammatory and coagulation parameters was lacking, thus the pathophysiological explanations for postoperative systemic responses remain primarily inferential and based on clinical observations. Multicenter prospective studies incorporating objective intraoperative perfusion assessments and comprehensive perioperative monitoring are necessary to further optimize individualized surgical decision-making for patients with PVG complicated by acute abdomen. Fourth, follow-up was limited to the in-hospital course and survival to discharge. Late bowel function and delayed complications after discharge could not be systematically assessed. Recurrent ischemia or reoperation was also not evaluated.

## Conclusion

Based on four cases, PVG appeared to mark serious intestinal pathology in patients with acute abdomen and supported timely surgical exploration. It did not, however, determine the need for bowel resection by itself. Intraoperative assessment of bowel viability may help guide individualized decisions between resection of clearly necrotic bowel and preservation of borderline ischemic segments. Bowel preservation may avoid unnecessary extensive resection, but it can increase postoperative management burden. These observations are exploratory and hypothesis-generating, and should be confirmed in larger studies.

## Data Availability

The raw data supporting the conclusions of this article will be made available by the authors, without undue reservation.

## Supplementary material

Supplementary Figure S1 Case-level representative preoperative CT findings of the four patients with PVG-associated acute abdomen. Each row represents one case, with three representative axial CT planes. White arrows indicate key radiological findings. **(A1–A3)** Case 1: intrahepatic portal venous gas, pneumatosis intestinalis, and mesenteric venous gas. **(B1–B3)** Case 2: intrahepatic portal venous gas, disordered small-bowel loops, and abnormal bowel configuration suggestive of internal hernia or small-bowel volvulus. **(C1–C3)** Case 3: intrahepatic portal venous gas on a cirrhotic liver background, bowel wall thickening, and a close-up view of branching intrahepatic portal venous gas. **(D1–D3)** Case 4: intrahepatic portal venous gas, bowel wall thickening with pneumatosis, and dilated or thickened bowel. All images were de-identified before inclusion. PVG, portal venous gas.
