# Supplementary figures and images for: Between definitive resection and damage control observation: a case series analysis of intraoperative decision-making for portal venous gas
Source: Front Surg. 2026 Jun 24;13:1853201. doi: 10.3389/fsurg.2026.1853201 (PMC13341660; doi:10.3389/fsurg.2026.1853201)

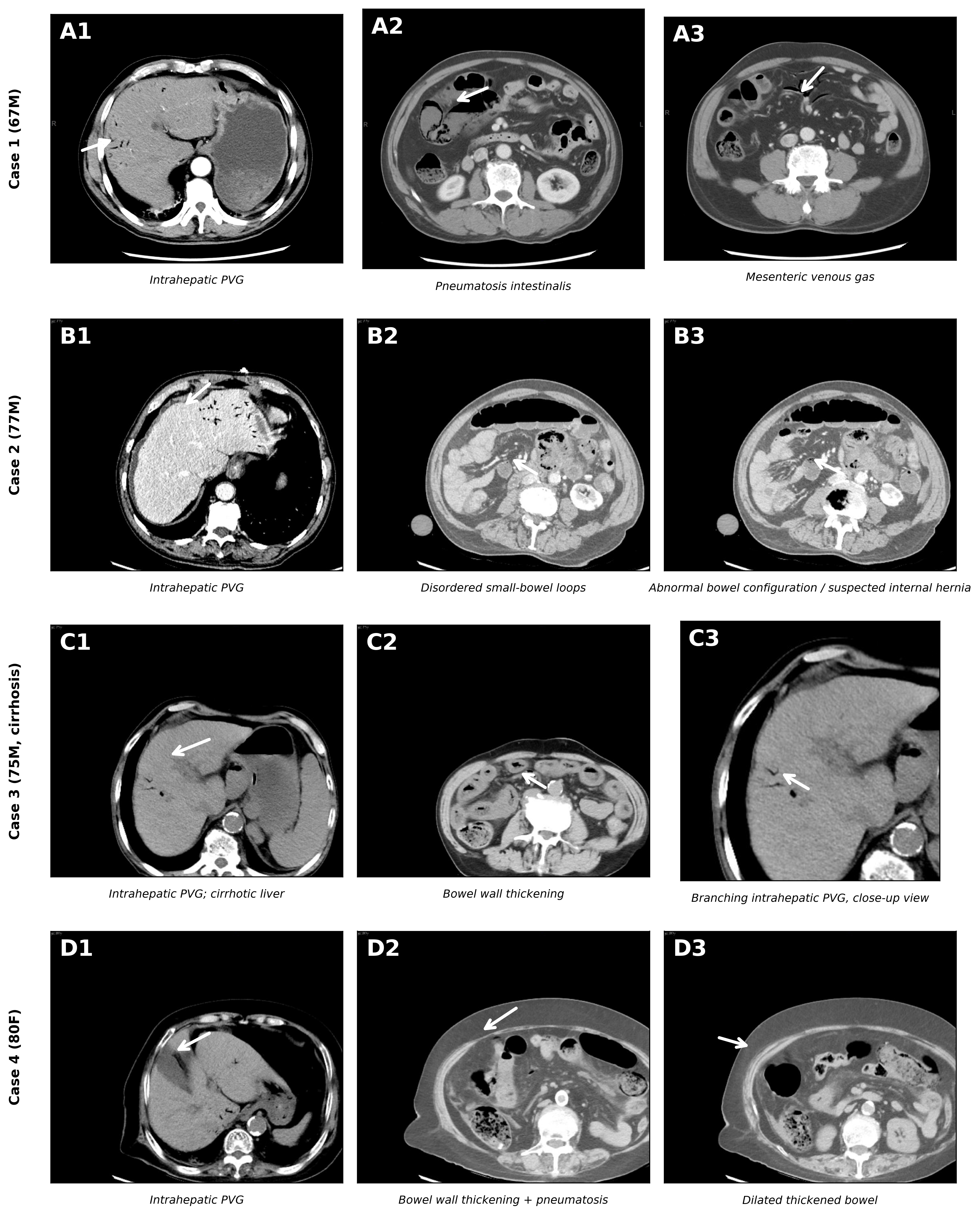

Supplement: Supplementary Figure S1 — Case-level representative preoperative CT findings of the four patients with PVG-associated acute abdomen. Each row represents one case, with three representative axial CT planes. White arrows indicate key radiological findings. (A1–A3) Case 1: intrahepatic portal venous gas, pneumatosis intestinalis, and mesenteric venous gas. (B1–B3) Case 2: intrahepatic portal venous gas, disordered small-bowel loops, and abnormal bowel configuration suggestive of internal hernia or small-bowel volvulus. (C1–C3) Case 3: intrahepatic portal venous gas on a cirrhotic liver background, bowel wall thickening, and a close-up view of branching intrahepatic portal venous gas. (D1–D3) Case 4: intrahepatic portal venous gas, bowel wall thickening with pneumatosis, and dilated or thickened bowel. All images were de-identified before inclusion. PVG, portal venous gas. [file Image1.tiff]
